# Sustainable implementation of practice-based research networks in primary care: a qualitative process evaluation of the Bavarian Research Practice Network (BayFoNet)

**DOI:** 10.1186/s12875-026-03459-3

**Published:** 2026-07-08

**Authors:** Linda Sanftenberg, Ulrike Stark-Felbinger, Stefanie Eck, Antonius Schneider, Peter Konstantin Kurotschka, Ildikó Gágyor, Stefanie Stark, Thomas Kühlein, Fabian Walter, Marco Roos, Tobias Dreischulte, Jochen Gensichen, the BayFoNet study group, Andrea Baumgärtel, Kathrin Lasher, Maike Ermster, Alexander Hapfelmeier, Susann Hueber, Merle Klanke, Christian Kretzschmann, Klaus Linde, Klara Lorenz, Til Uebel, Clara Teusen

**Affiliations:** 1 Institute of General Practice and Family Medicine, LMU University Hospital, LMU Medizin, Ludwig-Maximilians-Universität München, Nußbaumstr. 5, Munich, 80336 Germany; 2 Institute of General Practice and Health Services Research, Department Clinical Medicine, TUM School of Medicine and Health, Technical University of Munich (TUM), Munich, Germany; 3 Department of General Practice, University Hospital Würzburg, Würzburg, Germany; 4 Institute of General Practice, Friedrich-Alexander-University of Erlangen-Nuremberg, Erlangen, Germany; 5 General Practice, Medical Faculty, University of Augsburg, Augsburg, Germany

**Keywords:** Primary health Care, General practice, Practice based research networks, Implementation science, Participatory research

## Abstract

**Background:**

To expand research in general practice, the development of practice-based research networks (PBRNs) has increased considerably in Germany over the last years. In order to implement these structures sustainably and effectively in primary care, it is important to understand the perspective of the GP teams on their role and their experiences in conducting clinical studies within PBRNs. The aim of this analysis was to examine the experiences of GP teams after the implementation and conduct of clinical studies within in the Bavarian Practice-Based Research Network (BayFoNet) retrospectively to derive insights for a sustainable implementation of a German PBRN in primary care.

**Methods:**

We conducted semi-structured interviews based on the theoretical concept of the “Consolidated Framework for Implementation Research” with practices, which were already members of BayFoNet. The verbatim transcripts were analysed based on Kuckartz's qualitative content analysis.

**Results:**

Interviews were conducted with 16 GPs and one medical assistant (MA). As main drivers for a sustainable implementation of a primary care based PBRNs could be identified aspects of intervention characteristics (study design and a research question), the inner setting (teamwork), individual aspects (professional training) as well as facilitators on the level of the outer setting (contribution to evidence and quality). As barriers we identified a reduced interest of the study participants early processes (preparatory steps and participation) as well as missed networking options among peers (inner setting).

**Discussion:**

The perspective of the GP teams within our process evaluation confirmed some already known aspects, like the importance of professional development of the practice staff, contribution to evidence, quality and strength in general practice and networking needs. As increasing research-specific experience with clinical research sharpened their needs and requirements, desired processes of participation and the role of non-physician staff in PBRNs have to be further elaborated.

**Supplementary Information:**

The online version contains supplementary material available at 10.1186/s12875-026-03459-3.

## Introduction

Research in international primary care is crucial for high-quality patient care [1–3]. Even though a large proportion of all patients is cared for in the setting of general practice, only a small ratio of clinical trials is conducted in this setting [4]. Clinical studies are mainly conducted in university hospitals or research centres, and their results are usually not transferable to general practice [5, 6]. General practice provides significant research potential, given the large and diverse patient population, the wide range of symptoms, risk factors, and comorbidities encountered, the continuity of care for many chronically ill individuals, and the opportunities for implementing preventive measures [7]. To improve the quantity and quality of research in this setting, the collaboration between general practices and academic departments has to be strengthened with a sustainable implementation of practice-based research networks (PBRNs) [8]. PBRNs provide a vital link between academic general practice and practices to enable a practice-theory–practice loop and contribute to evidence-based quality improvement [6, 9–13]. They might improve cooperation and exchange between various stakeholders in the healthcare system and politics, support further training of general practitioners (GPs) and strengthen patient and public involvement (PPI) in clinical research [14]. GP practice teams not only enable patient recruitment, but also clinical research under real-world conditions and development of practice-relevant research questions. PBRNs create the conditions for general practice to generate its own evidence—closely aligned with clinical patient care—rather than having to adopt evidence derived from specialized or inpatient settings. Collaboration in PBRNs not only strengthens research, but also the further training of GPs and their practice staff and the implementation of new findings into patient care. The involvement of GP practice teams in clinical research has helped to improve patient care and ensures that patients' needs are better reflected in research [15]. While other countries such as the USA, Canada, the UK and the Netherlands have a well-developed structure of PBRNs, the history of German PBRNs is comparatively young [16–22].

Since 2020, the German Federal Ministry of Education and Research (BMBF) supports the establishment of a sustainable network structure for GP practices in Germany by funding regional network centres (RNCs), such as the Bavarian Practice-Based Research Network (BayFoNet).

GP practices had to obtain at least a basic qualification to conduct clinical studies to be accredited in BayFoNet. Courses to achieve this level of basic qualification are organized by the coordination office of BayFoNet and provided as regular web-based trainings [23]. GP practices who are already collaborating with the RNCs in teaching or primary care research are invited to participate as partners in BayFoNet by invitations letters [24]. These online courses are held five times per year and cover a clinically relevant topic of daily patient care, as well as a topic on research methodology in primary care. Furthermore, every three months there are in-person meetings with GP teams (`think tank`) to discuss basic study ideas, develop clinical trials in a participatory manner and to disseminate study results into practice.

To elaborate the development of clinicals research in general practice and the sustainable implementation of BayFoNet, the RNC at the LMU Munich is currently performing a mixed-methods process evaluation (PE) at three different time points [24].

The first step of this process evaluation was conducted prior to the implementation of two pilot cluster-randomized trials in GP practices to derive insights for the sustainable implementation of a German PBRN in primary care (BayFoNet) [25]. The second evaluation step occurred during the implementation of these pilot studies [26]. This article reports the third evaluation step, which retrospectively and qualitatively analysed the experiences of GP teams after their participation in the cluster randomized controlled trials.

## Material and methods

### Study design

**Fig. 1:**
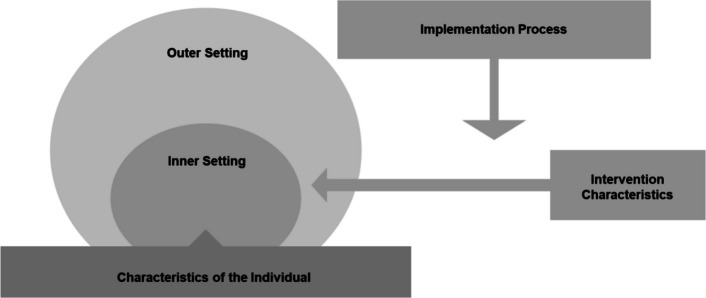
Diagrammatic overview of the Consolidated Framework for Implementation Research (CFIR) 2.0 (modified due to [27])

A semi-structured interview guide was developed based on the theoretical concept of the “Consolidated Framework for Implementation Research” (CFIR). The CFIR is an established and structured framework to evaluate implementation factors in the context of primary care. Five overarching domains are considered within the CFIR, namely Intervention Characteristics, Outer Setting, Inner Setting, Characteristics of the Individual and Implementation Process (online Supplemental file 1) [27] (Fig. 1).

The interview guide contained two main sections. While the first part referred to the implementation of clinical studies in daily general practice, the second part referred to a sustainable implementation of BayFoNet. The following subjects were addressed: personal interest in clinical research and active study implementation, as well as previous experiences with clinical research. We asked for barriers and facilitators for conducting clinical research in general practice. Furthermore, we evaluated needs and preferences for a further development of BayFoNet network (online Supplemental file 2). Prior to data collection, a pilot interview was conducted with a physician in training at the Institute for General Practice and Family Medicine at the LMU Munich.

### Sampling and recruitment

We invited a convenience sample of BayFoNet-accredited GP practices. The first written invitation for study participation was sent by email to all GP practices in July 2024, with two additional written reminders sent out in August and September 2024.

The calls explicitly addressed all professional groups of the GP teams, including both physicians as well as medical assistants (MAs). In addition, a compensation for expenses of 100 euros was announced as a financial incentive for each study participant independent from the professional qualification.

### Data collection

The interviews were conducted by telephone, video call or in person, depending on the participants' preference. Only the study participant and the researcher were present during the actual interview. The interviewing researcher was a medical student who conducted the qualitative study interviews in a self-reflective, neutral manner (US-F). At the beginning of each interview, the interviewer introduced herself as a medical student and explained that the results of the interviews would be used for her doctoral thesis. The transcripts were not given to the interviewees for comment or correction, due to methodological and practical considerations. The time delay between interviews and feedback may lead to reconstructed responses, and transcribed interviews may not be perceived as an authentic representation of spoken language, potentially prompting revisions. In addition, retrospective validation can introduce socially desirable or post-hoc rationalized answers that may alter the original data. Instead, we ensured rigor through a systematic qualitative content analysis following Kuckartz and a theory-informed framework using the CFIR. The interviews were digitally recorded as audio files, transcribed verbatim and pseudonymized for the analysis. Most of the interviews were conducted in parallel with recruitment. None of the interviews had to be repeated or excluded from the data analysis. When the final interviews revealed no additional areas and no further variance within the themes, data saturation was assumed. Data saturation was applied as a pragmatic and well-established stopping criterion appropriate for our relatively focused, homogeneous sample and structured CFIR-informed qualitative content analysis, which allowed iterative assessment of thematic redundancy [28].

### Data analysis

The collected data was analysed based on Kuckartz's qualitative content analysis, whereby deductive and inductive categories were formed [29]. We chose qualitative content analysis over reflexive thematic analysis because our aim was a systematic, CFIR-guided categorisation of implementation barriers and facilitators rather than an interpretative, latent theme development, allowing for transparent deductive–inductive coding aligned with a predefined theoretical framework. The text passages were coded, ordered, further substantiated and systematized with the help of the MAXQDA 2020 software program. In addition, inductive categories were formed by reading the entire data set several times. The reporting to the qualitative analysis followed the COREQ (COnsolidated criteria for REporting Qualitative research) checklist (online Supplemental file 3) [30].

## Results

### Characteristics of the study population

A total of 16 GPs and one MA from 17 different GP practices agreed to be interviewed between July to October 2024. Half of the interviewed GPs were male (*n* = 8), the MA was female. Most of the interviewed GPs (*n* = 14) held a medical doctorate.

The characteristics of all analysed GP practices are shown in Table 1.

**Table 1:** Characteristics of the analysed GP practices

GP practice location:	Rural GP practice *n* = 14 (< 100.000 inhabitants)	Urban GP practice *n* = 3 (> 100.000 inhabitants)
GP practice structure:	Joint GP practice* n* = 8	Single GP practice *n* = 9
GP practice already affiliated as teaching practice:	Yes *n* = 8	No *n* = 9

The interviews were conducted via video conference (*n* = 8), telephone call (*n* = 7) or in person (*n* = 2). The duration of the individual interviews varied between 22–61 min.

### Barriers and facilitators for a sustainable implementation of PBRNs in primary care

The interviews revealed a range of barriers and facilitators relevant to the sustainable implementation of PBRNs in primary care. Main drivers for a successful implementation of a PBRN in primary care were corresponding to the CFIR domains “intervention characteristics”, “inner setting” and “outer setting”. Clinical studies that are introduced, should offer a study design that is easy to conduct answering relevant questions of daily patients care (“intervention characteristics”). Team-based approaches should be addressed (“inner setting”), whereas the overarching goal of participating in a primary care based PBRN was a contribution to evidence, strength and quality within the discipline of general practice (“outer setting”).

On the other hand, cited barriers mainly referred to the CFIR domains “process” and “inner setting”. Besides a lack of interest to participate in early phases of study design and preparatory steps (“process”), study participants emphasized missing networking options with the PBRN. (“inner setting”) An overview of all identified themes is provided in Table 2.

**Table 2:** Identified barriers and facilitators according to CFIR [27]

Main facilitators	Main barriers
Characteristics of the intervention: simple study design; relevant research question (*n* = 16)	Process: lack of interest in early participation and preparatory steps (*n* = 14)
Inner setting: teamwork within the GP practice (*n* = 11)	Inner setting: lack of networking with peers (*n* = 8)
Individuals: professional development and further training of practice staff (*n* = 17)	
Outer setting: contribution to evidence, strength and quality within the discipline of general practice (*n* = 12)	

The following presentation of results is based on the interview guide (see online Supplemental file 1). “(…)” means a break in the narrative flow, “[…]” means a shortening of the quote.

#### Simple study design with a relevant research question for daily patient care

First of all, most study participants emphasized that the research question of a clinical trial should be of perceived relevance to everyday patient care and to the field of the GPs specialization to increase attraction.

“Studies that actually relate to my field of treatment are attractive. So, if I can see that I can learn something from it and that there is a direct benefit for my patients, yes.” (B7_Transcript_audio1649684324.m4a, Pos. 3).

Furthermore, many interview partners emphasized the importance of the intervention design, including practicability in everyday practice, including resource saving processes. They highly appreciated the ongoing support of the RNCs.

“The time required is important. But the support in the preparation of the study was given, which is also very important.” (B3_Transcript_audio1072472036.m4a, Pos. 73)

#### Teamwork within the GP practice

Beside ongoing support of the academic RNCs, most of the study participant identified team work as another main facilitator to ensure a successful implementation of study-specific processes. Involvement of physicians as well as non-physicians in different tasks seems to be essential to cope with the additional work in daily practice.

“Involving the MAs is a relief in any case, exactly.” (B9_Transcript_ds260203.mp3, Pos. 23)

“Of course, I then also involved my colleagues and said: “Hey, if you hear that a patient is calling with these symptoms […] please call them in the same day or the next day, because maybe we could include them in the study. Without my colleagues, we would not be able to successfully manage patient care and research simultaneously. (B14_Transcript_ds260208.mp3, Pos. 34).

#### Professional development and further training of practice staff

As BayFoNet includes regular training series, GP team members perceived an increase in knowledge and skills both for themselves and their colleagues as potential facilitators to actively engage in a primary care based PBRN. The study participants had a strong internal drive to reflect on and improve their own practices, as well as to continuously develop both their own abilities and those of their GP team.

“[…] and very easily you get more skills and knowledge and new suggestions for an improved everyday practice” (B8_Transcript_ds260202.mp3, Pos. 95)

#### Contribution to evidence, strength and quality in general practice

Another driver for a sustainable implementation of PBRNs in primary care is the potential to enhance the reputation of primary care in society and health care politics, as well as to contribute to evidence, strength and quality in general practice. In particular, GPs want to strengthen the image of general practice in order to obtain and retain new trainees as successors.

“I like being a rural physician and I love being a GP. But I also want to get rid of this fairy-tale image in general practice. Because that's not how we get trainees. […] And we have to make progress in medicine and we need young talents. […] And that's where I think BayFoNet is actually a really good story.” (B13_Transcript_ds260207.mp3, Pos. 69).

#### Lack of interest in early participation

However, the majority of the study participants was not interested in active involvement in early phases of study design or preparatory steps that are needed to plan and develop clinical trials. They were satisfied to receive invitations from the RNCs on regular basis and to select the studies according to their personal interest and time capacities.

I:”[…] If engagement in early phases of study design is not of particular interest for you: how would you like to participate in the future?”

B:” It's good the way it is. I'm waiting for e-mails that there's something that suits us (laughs) and that something will come about again.”

(B17_Transcript_ds1412606228.mp3, Pos. 91–92)

#### Lack of networking with peers

It was striking that, while contact with the RNCs was perceived as positive and beneficial, almost half of the interviewed GP team members reported missing contact with other GP team members within the network,

*“No, you don't really notice the network. The only contact is the department you work with, which is important. I had no contact at all with any other GPs.”*

(B4_Transcript_ds1995791352.mp3, Pos. 109)

## Discussion

### Statement of principal findings

Key facilitators a sustainable implementation of a German PBRN referred mainly to the characteristics of the intervention itself. To implement clinical studies in GP practices, study designs should be easy to conduct and research question should focus on subjects with high relevance for the daily work of GP teams in patient care (“intervention characteristics”). All interviewed GP team members (physicians as well as non-physicians) highlighted the importance of teamwork within their practice (“inner setting”) to ensure the success of study implementation besides patient care. Another important driver on the level of the individual GP team members (“individuals”) for a sustainable implementation of a German PBRN were the possibility to improve professional competences and knowledge to attain improved patient care in the long-term. With their active participation in a primary care based PBRN, the study participants want to improve the role and image of general practice within society and health care service, especially to achieve a sufficient number of residents for the future (“outer setting”). Contradictory, an early involvement in the development of research questions and study designs was not desired (“process”). As a key barrier, the missing regular networking with peers was pronounced (“inner setting”).

### Comparison to literature

The results of our study confirm many insights from other international PBRNs. Nevertheless, the current study elucidated specific details regarding known determinants particularly through the comparison of three different time points within our process evaluation.

There is a number of studies which indicate, that the research question as well as the design of a clinical study are of the utmost importance for establishing PBRNs in primary care [31, 32]. Pioneering GP practices exhibited already in the 1970 s defining characteristics for PBRNs like research questions focused on optimal care for patients seen in community practices, appreciation for the need to do research without disrupting or distorting the practices’ purpose as well as enthusiasm for simple data collection [22].

During the first two steps of our process evaluation, the importance of teamwork was cited and a lack of motivation among MAs was identified as a main barrier to implement ongoing clinical trials [26]. In contrast to the evaluation during the implementation of both cluster-randomized pilot trials (*n* = 15 MAs; [26]), there was only a single MA who responded to our invitation after the interventional studies had been conducted. It can be concluded, that MAs seemed to interpret their role in the research process as purely executive during the interventional phase. However, there is evidence, that a collaborative approach is essential for the successful conduct of clinical trials and the advancement of medical research [33]. Furthermore, an increased involvement of MAs in clinical research and task delegating might have very positive effects on their job satisfaction and improved patient care [34–36].

Besides collaboration, professional development and further training of the practice staff has already been emphasized in different contexts as a strong motivator to actively engage in PBRNs and to ensure the quality of study performance [25, 33, 37]. It can be assumed, that offering regular qualification levels will guarantee a sustainable implementation of PBRNs in primary care, since an essential part of the medical profession is continuous further training and lifelong learning [38–41].

Furthermore, contribution to evidence, quality and strength in general practice was identified as a main facilitator for the development and implementation of different primary care-based PBRNs [16, 22, 25, 26]. However, during the presented analysis we were able to identify another important detail regarding this facilitator: not only to increase the reputation of general practice within society and health politics, but to make general practice attractive for potential postgraduate trainees or even clinician scientists [42–44].

Clinical research should emerge from daily patient care and highly relevant research questions should be developed [22, 45]. Accordingly, BayFoNet has launched regular think tanks, where GPs are invited to share their research ideas and to develop their own research projects with support from the academic institutions [46]. After completion of both interventions, some GPs stated that they had too few resources for early involvement in the developmental stages of potential research projects. Other GPs were simply not interested in a co-designing role and preferred to accompany the implementation of proposed studies. This development may be explained by the fact that a better understanding of research-relevant content simultaneously renders both the complexity of the research process and the associated time commitment more comprehensible [47, 48].

Networking with peers and academic departments is an already known facilitator for the successful implementation of PBRNs in primary care [17, 22, 32]. However, contact with peers remains sparse in many PBRNs, despite regular options to meet online as well as personally [26, 49]. The discrepancy between the desired networking with peers and the lack of utilisation of regular in-person as well as online invitations should be further investigated. To strengthen networking with peers, it might be helpful to offer meetings with a reference to a clinical research question for focused exchange [50]. The discrepancy should be resolved, as networking with peers enables the exchange of experiences, fosters learning from each other and represents an essential component of a PBRN [9, 51–53].

### Strengths and limitations of the study

This study provides important insights into the experiences and role understandings of accredited GP teams of BayFoNet, as well as their needs and preferences for a sustainable implementation of primary care based PBRNs in Germany. Results might be transferable to national as well as international PBRNs and could be considered for the preparation of future clinical studies in primary care.

Due to logistical and temporal constraints, finalized interpretations and emergent themes were not returned to the study participants for review. To mitigate this limitation, the research team maintained strict reflexivity, kept detailed memos, and anchored all interpretations directly to verbatim participant quotes. Future studies should endeavor to incorporate respondent validation to further corroborate the accuracy and resonance of the qualitative analysis. As a limitation regarding the generalizability of the results, it has to be mentioned that approximately 12,000 GPs were registered 2024 in Bavaria, Germany. During this period, around n = 260 GP teams were officially affiliated with BayFoNet, representing approximately 2% of all GPs in Bavaria. Furthermore, the participating GPs are already members of an PBRN, which makes them an highly selected group. The findings therefore are mainly applicable to GPs interested in research, and not necessarily mainstream GPs without any connection to academia.

## Conclusions

PBRNs have gained importance in recent years and have made a valuable contribution to the advancement of patients care. Research questions with high relevance for everyday practice and feasible study designs of clinical trials based on teamwork were identified as main facilitators as well as an ongoing professional training for all staff members. Aspects such as reduced interest in early participation in study design, insufficient networking withs peers and the role of non-physician staff in PBRNs should be addressed in the future development of PBRNs in primary care to ensure their sustainable implementation.

## Supplementary Information

Supplementary Material 1: Framework Guidance for the “Consolidated Framework for Implementation Research (CFIR)”.

Supplementary Material 2: Interview guide for GP teams.

Supplementary Material 3: Consolidated criteria for reporting qualitative research (COREQ).

## Data Availability

Data and materials might be obtained from the authors upon reasonable request.

## Abbreviations

BayFoNet: Bavarian Research Practice Network
BMBF: Federal Ministry of Education and Research
CFIR: Consolidated Framework for Implementation Research
GP: General practitioner
MA: Medical assistant
PBRN: Practice-based research network
PE: Process evaluation
PPI: Patient and Public Involvement
RNC: Regional network centers
