# Supplementary material for: Sustainable implementation of practice-based research networks in primary care: a qualitative process evaluation of the Bavarian Research Practice Network (BayFoNet)
Source: BMC Prim Care. 2026 Jul 8;27:261. doi: 10.1186/s12875-026-03459-3 (PMC13355365; doi:10.1186/s12875-026-03459-3)
Supplement: Supplementary file 1 — Supplementary Material 1: Framework Guidance for the “Consolidated Framework for Implementation Research (CFIR)”. [file 12875_2026_3459_MOESM1_ESM.pdf]

Framework Guidance:
The CFIR is intended to be used to collect data from individuals who have power and/or influence over implementation outcomes. See the CFIR Outcomes Addendum for guidance on identifying these individuals and selecting outcomes.
The CFIR must be fully operationalized prior to use in a project:
1) Define the subject of each domain for the project (see guidance for each domain below).
2) Replace broad construct language with project-specific language if needed.
3) Add constructs to capture salient themes not included in the updated CFIR.

I. INNOVATION DOMAIN	
**Construct Name**	**Construct Definition**<br>*The degree to which:*
A. Innovation Source	The group that developed and/or visibly sponsored use of the innovation is reputable, credible, and/or trustable.
B. Innovation Evidence-Base	The innovation has robust evidence supporting its effectiveness.
C. Innovation Relative Advantage	The innovation is better than other available innovations or current practice.
D. Innovation Adaptability	The innovation can be modified, tailored, or refined to fit local context or needs.
E. Innovation Trialability	The innovation can be tested or piloted on a small scale and undone.
F. Innovation Complexity	The innovation is complicated, which may be reflected by its scope and/or the nature and number of connections and steps.
G. Innovation Design	The innovation is well designed and packaged, including how it is assembled, bundled, and presented.
H. Innovation Cost	The innovation purchase and operating costs are affordable.
**II. OUTER SETTING DOMAIN**	
**Construct Name**	**Construct Definition**<br>*The degree to which:*
A. Critical Incidents	Large-scale and/or unanticipated events disrupt implementation and/or delivery of the innovation.
B. Local Attitudes	Sociocultural values (e.g., shared responsibility in helping recipients) and beliefs (e.g., convictions about the worthiness of recipients) encourage the Outer Setting to support implementation and/or delivery of the innovation.
C. Local Conditions	Economic, environmental, political, and/or technological conditions enable the Outer Setting to support implementation and/or delivery of the innovation.
D. Partnerships & Connections	The Inner Setting is networked with external entities, including referral networks, academic affiliations, and professional organization networks.
E. Policies & Laws	Legislation, regulations, professional group guidelines and recommendations, or accreditation standards support implementation and/or delivery of the innovation.
F. Financing	Funding from external entities (e.g., grants, reimbursement) is available to implement and/or deliver the innovation.
G. External Pressure	External pressures drive implementation and/or delivery of the innovation. Note: Use this construct to capture themes related to External Pressures that are not included in the subconstructs below.
1. Societal Pressure	Mass media campaigns, advocacy groups, or social movements or protests drive implementation and/or delivery of the innovation.
2. Market Pressure	Competing with and/or imitating peer entities drives implementation and/or delivery of the innovation.
3. Performance-Measurement Pressure	Quality or benchmarking metrics or established service goals drive implementation and/or delivery of the innovation.
**III. INNER SETTING DOMAIN**	
**Construct Name**	**Construct Definition**<br>*The degree to which:*
Note:	*Constructs A – D exist in the Inner Setting regardless of implementation and/or delivery of the innovation, i.e., they are persistent general characteristics of the Inner Setting.*
A. Structural Characteristics	Infrastructure components support functional performance of the Inner Setting. Note: Use this construct to capture themes related to Structural Characteristics that are not included in the subconstructs below.
1. Physical Infrastructure	Layout and configuration of space and other tangible material features support functional performance of the Inner Setting.
2. Information Technology Infrastructure	Technological systems for tele-communication, electronic documentation, and data storage, management, reporting, and analysis support functional performance of the Inner Setting.
3. Work Infrastructure	Organization of tasks and responsibilities within and between individuals and teams, and general staffing levels, support functional performance of the Inner Setting.

B. Relational Connections	There are high quality formal and informal relationships, networks, and teams within and across Inner Setting boundaries (e.g., structural, professional).
C. Communications	There are high quality formal and informal information sharing practices within and across Inner Setting boundaries (e.g., structural, professional).
D. Culture	There are shared values, beliefs, and norms across the Inner Setting. Note: Use this construct to capture themes related to Culture that are not included in the subconstructs below.
1. Human Equality-Centeredness	There are shared values, beliefs, and norms about the inherent equal worth and value of all human beings.
2. Recipient-Centeredness	There are shared values, beliefs, and norms around caring, supporting, and addressing the needs and welfare of recipients.
3. Deliverer-Centeredness	There are shared values, beliefs, and norms around caring, supporting, and addressing the needs and welfare of deliverers.
4. Learning-Centeredness	There are shared values, beliefs, and norms around psychological safety, continual improvement, and using data to inform practice.
Note:	*Constructs E – K are specific to the implementation and/or delivery of the innovation.*
E. Tension for Change	The current situation is intolerable and needs to change.
F. Compatibility	The innovation fits with workflows, systems, and processes.
G. Relative Priority	Implementing and delivering the innovation is important compared to other initiatives.
H. Incentive Systems	Tangible and/or intangible incentives and rewards and/or disincentives and punishments support implementation and delivery of the innovation.
I. Mission Alignment	Implementing and delivering the innovation is in line with the overarching commitment, purpose, or goals in the Inner Setting.
J. Available Resources	Resources are available to implement and deliver the innovation. Note: Use this construct to capture themes related to Available Resources that are not included in the subconstructs below.
1. Funding	Funding is available to implement and deliver the innovation.
2. Space	Physical space is available to implement and deliver the innovation.
3. Materials & Equipment	Supplies are available to implement and deliver the innovation.
K. Access to Knowledge & Information	Guidance and/or training is accessible to implement and deliver the innovation.
**IV. INDIVIDUALS DOMAIN**	
**ROLES SUBDOMAIN**	
**Construct Name**	**Construct Definition**
A. High-level Leaders	Individuals with a high level of authority, including key decision-makers, executive leaders, or directors.
B. Mid-level Leaders	Individuals with a moderate level of authority, including leaders supervised by a high-level leader and who supervise others.
C. Opinion Leaders	Individuals with informal influence on the attitudes and behaviors of others.
D. Implementation Facilitators	Individuals with subject matter expertise who assist, coach, or support implementation.
E. Implementation Leads	Individuals who lead efforts to implement the innovation.
F. Implementation Team Members	Individuals who collaborate with and support the Implementation Leads to implement the innovation, ideally including Innovation Deliverers and Recipients.
G. Other Implementation Support	Individuals who support the Implementation Leads and/or Implementation Team Members to implement the innovation.
H. Innovation Deliverers	Individuals who are directly or indirectly delivering the innovation.
I. Innovation Recipients	Individuals who are directly or indirectly receiving the innovation.
**CHARACTERISTICS SUBDOMAIN**	
**Construct Name**	**Construct Definition:**<br>*The degree to which:*
A. Need	The individual(s) has deficits related to survival, well-being, or personal fulfillment, which will be addressed by implementation and/or delivery of the innovation.
B. Capability	The individual(s) has interpersonal competence, knowledge, and skills to fulfill Role.
C. Opportunity	The individual(s) has availability, scope, and power to fulfill Role.
D. Motivation	The individual(s) is committed to fulfilling Role.

V. IMPLEMENTATION PROCESS DOMAIN	
**Construct Name**	**Construct Definition**<br>*The degree to which individuals:*
A. Teaming	Join together, intentionally coordinating and collaborating on interdependent tasks, to implement the innovation.
B. Assessing Needs	Collect information about priorities, preferences, and needs of people. Note: Use this construct to capture themes related to Assessing Needs that are not included in the subconstructs below.
1. Innovation Deliverers	Collect information about the priorities, preferences, and needs of deliverers to guide implementation and delivery of the innovation.
2. Innovation Recipients	Collect information about the priorities, preferences, and needs of recipients to guide implementation and delivery of the innovation.
C. Assessing Context	Collect information to identify and appraise barriers and facilitators to implementation and delivery of the innovation.
D. Planning	Identify roles and responsibilities, outline specific steps and milestones, and define goals and measures for implementation success in advance.
E. Tailoring Strategies	Choose and operationalize implementation strategies to address barriers, leverage facilitators, and fit context.
F. Engaging	Attract and encourage participation in implementation and/or the innovation. Note: Use this construct to capture themes related to Engaging that are not included in the subconstructs below.
1. Innovation Deliverers	Attract and encourage deliverers to serve on the implementation team and/or to deliver the innovation.
2. Innovation Recipients	Attract and encourage recipients to serve on the implementation team and/or participate in the innovation.
G. Doing	Implement in small steps, tests, or cycles of change to trial and cumulatively optimize delivery of the innovation.
H. Reflecting & Evaluating	Collect and discuss quantitative and qualitative information about the success of implementation. Note: Use this construct to capture themes related to Reflecting & Evaluating that are not included in the subconstructs below.
1. Implementation	Collect and discuss quantitative and qualitive information about the success of implementation.
2. Innovation	Collect and discuss quantitative and qualitative information about the success of the innovation.
I. Adapting	Modify the innovation and/or the Inner Setting for optimal fit and integration into work processes.

CFIR OUTCOMES ADDENDUM<br>I. ANTECEDENT ASSESSMENTS	
**Name**	**Definition**
A. Acceptability	The extent to which an innovation is perceived as “agreeable, palatable, or satisfactory" (Proctor, 2009).
B. Appropriateness	The “perceived fit, relevance, or compatibility of the innovation […] for a given practice setting, provider, or consumer; and/or perceived fit of the innovation to address a particular issue or problem" (Proctor, 2009).
C. Feasibility	The extent to which an innovation “can be successfully used or carried out within a given agency or setting" (Proctor, 2009).
D. Implementation Climate	The extent to which the Inner Setting has an implementation climate.
E. Implementation Readiness	The extent to which the Inner Setting is ready for implementation.

II. IMPLEMENTATION OUTCOMES	
**Name**	**Definition**
A. Anticipated Implementation Outcomes	Outcomes based on perceptions or measures of the likelihood of future implementation success or failure, i.e., implementation outcomes that have not yet occurred. These outcomes are forward-looking; constellations of CFIR determinants across domains predict these outcomes.
1. Adoptability	The likelihood key decision-makers will decide to put the innovation in place/innovation deliverers will decide to deliver the innovation.
2. Implementability	The likelihood the innovation will be put in place or delivered.
3. Sustainability	The likelihood the innovation will be put in place or delivered over the long-term.
B. Actual Implementation Outcomes	Outcomes based on perceptions or measures of current (or past) implementation success or failure, i.e., implementation outcomes that have occurred. These outcomes are backward-looking; constellations of CFIR determinants across domains explain these outcomes.
1. Adoption	The extent key decision-makers decide to put the innovation in place/innovation deliverers decide to deliver the innovation.
2. Implementation	The extent the innovation is in place or being delivered.
3. Sustainment	The extent the innovation is in place or being delivered over the long-term.

III. INNOVATION OUTCOMES	Outcomes that capture the success or failure of the innovation, based on the impact of the innovation on three important constituents: Innovation Recipients, Innovation Deliverers, and Key Decision-Makers. Impact is defined by: Reach ("The absolute number, proportion, and representativeness of individuals who are willing to participate in a given initiative, intervention, or program") x Innovation Effectiveness ("The impact of an intervention on important outcomes, including potential negative effects, quality of life, and economic outcomes" (Glasgow et al. 2019).
**Name**	**Definition**
A. Innovation Recipient Impact	Recipient Reach x Innovation Effectiveness
B. Innovation Deliverer Impact	Deliverer Reach x Innovation Effectiveness
C. Key-Decision Maker (or System) Impact	Key-Decision Maker Reach x Innovation Effectiveness
