# Supplementary material for: Sustainable implementation of practice-based research networks in primary care: a qualitative process evaluation of the Bavarian Research Practice Network (BayFoNet)
Source: BMC Prim Care. 2026 Jul 8;27:261. doi: 10.1186/s12875-026-03459-3 (PMC13355365; doi:10.1186/s12875-026-03459-3)
Supplement: Supplementary file 2 — Supplementary Material 2: Interview guide for GP teams. [file 12875_2026_3459_MOESM2_ESM.pdf]

<online supplemental file 2: Interview guide for the general practitioners>

Part 1: Implementation factors of clinical studies in GP practices

CFIR Domain	
Innovation Characteristics	• Why do you (want to) conduct clinical trials in your everyday practice?<br>• Based on your experience: What makes conducting clinical trials attractive for GP practices?
Outer Setting	• Which external barriers and facilitators did you perceive for the implementation of clinical studies in your practice?
Inner Setting	• In retrospect: has the active implementation of a clinical study changed anything in your own practice?<br>• Which internal barriers and facilitators did you perceive for the implementation of clinical studies?
Characteristics of the Individual	• What kind of study designs do you prefer to implement in your practice?<br>• Are there any research questions that are of particular interest to you?
Implementation Process	• What preparations have you made to be able to better conduct clinical trials in your practice in the future?

Part 2: Determinants for a sustainable implementation of the PBRN “BayFoNet”

CFIR Domaine	
Intervention Characteristics	• What makes participation in BayFoNet attractive for GP practices?
Inner Setting	• How should BayFoNet be improved in the future to create a sustainable benefit for clinical research in GP practices?<br>• Did you perceive BayFoNet as a lively network that promotes the exchange with the academic general practice and other GP practices?
Implementation Process	• What should this participation look like in the future?
